# Correlation Between Fecal Microbiota and Corticosteroid Responsiveness in Primary Immune Thrombocytopenia: an Exploratory Study

**DOI:** 10.1002/advs.202410417

**Published:** 2025-03-05

**Authors:** Feng‐Qi Liu, Zhuo‐Yu An, Li‐Juan Cui, Meng‐Yu Xiao, Ye‐Jun Wu, Wei Li, Bang‐Shuo Zhang, Li Yu, Jia Feng, Zhuo‐Gang Liu, Ru Feng, Zhong‐Xing Jiang, Rui‐Bin Huang, Hong‐Mei Jing, Jin‐Hai Ren, Xiao‐Yu Zhu, Yun‐Feng Cheng, Yu‐Hua Li, He‐Bing Zhou, Da Gao, Yi Liu, Fan Yu, Xin Wang, Jian‐Lin Qiao, Dai‐Hong Hu, Lu‐Lu Wang, Meng‐Tong Zang, Qi Chen, Qing‐Yuan Qu, Jian‐Ying Zhou, Meng‐Lin Li, Yu‐Xiu Chen, Qiu‐Sha Huang, Hai‐Xia Fu, Yue‐Ying Li, Qian‐Fei Wang, Xiao‐Jun Huang, Xiao‐hui Zhang, the Cooperative ITP Working Group

**Affiliations:** ^1^ Peking University People's Hospital, Peking University Institute of Hematology, National Clinical Research Center for Hematologic Disease, Beijing Key Laboratory of Hematopoietic Stem Cell Transplantation Collaborative Innovation Center of Hematology Beijing 100044 China; ^2^ Department of Hematology The General Hospital of Ningxia Medical University Yinchuan 750004 China; ^3^ Department of Hematology The First Bethune Hospital of Jilin University Changchun 130021 China; ^4^ Department of Hematology Three Gorges Hospital Affiliated to Chongqing University Chongqing 404000 China; ^5^ Department of Hematology The Second Affiliated Hospital of Nanchang University Nanchang 330008 China; ^6^ Department of Hematology Peking University Shenzhen Hospital Shenzhen 518036 China; ^7^ Department of Hematology Shengjing Hospital of China Medical University Shenyang 110136 China; ^8^ Department of Hematology Beijing Hospital Beijing 100005 China; ^9^ Department of Hematology First Affiliated Hospital of Zhengzhou University Zhengzhou 450000 China; ^10^ Department of Hematology The First Affiliated Hospital of Nanchang University Nanchang 210029 China; ^11^ Department of Hematology Lymphoma Research Center Peking University Third Hospital Beijing 100191 China; ^12^ Department of Hematology Second Hospital of Hebei Medical University Shijiazhuang 050000 China; ^13^ Department of Hematology, The First Affiliated Hospital of USTC, Division of Life Sciences and Medicine University of Science and Technology of China Hefei 230001 China; ^14^ Department of Hematology Zhongshan Hospital Fudan University Shanghai 200032 China; ^15^ Department of Hematology Zhujiang Hospital of Southern Medical University Guangzhou 510280 China; ^16^ Department of Hematology Beijing Luhe Hospital Capital Medical University Beijing 101199 China; ^17^ Department of Hematology The Affiliated Hospital of Inner Mongolia Medical University Hohhot 010050 China; ^18^ Department of Hematology Senior Department of Hematology the Fifth Medical Center of PLA General Hospital Beijing 100048 China; ^19^ Department of Hematology Tsinghua Changgung Hospital Beijing 102218 China; ^20^ Department of Hematology Shandong Provincial Hospital Affiliated to Shandong First Medical University Jinan 250021 China; ^21^ Department of Hematology The Affiliated Hospital of Xuzhou Medical University Xuzhou 221000 China; ^22^ CAS Key Laboratory of Genomic and Precision Medicine Beijing Institute of Genomics Chinese Academy of Sciences and China National Center for Bioinformation Beijing 100101 China; ^23^ University of Chinese Academy of Sciences Beijing 100101 China; ^24^ Peking‐Tsinghua Center for Life Sciences, Academy for Advanced Interdisciplinary Studies Peking University Beijing 100074 China; ^25^ State Key Laboratory of Natural and Biomimetic Drugs Peking University Beijing 100191 China

**Keywords:** biomarker, gut microbiome, gut microbiota, immune thrombocytopenia, machine learning

## Abstract

Corticosteroids (CSs) are the initial therapy for immune thrombocytopenia (ITP); however, their efficacy is not adequately predicted. As a novel biomarker, the composition of the gut microbiota is non‐invasively tested and altered in patients with ITP. This study aims to develop a predictive model that leverages gut microbiome data to predict the CS response in patients with ITP within the initial four weeks of treatment. Metagenomic sequencing is performed on fecal samples from 212 patients with ITP, 152 of whom underwent CS treatment and follow‐up. Predictive models are trained using six machine‐learning algorithms, integrating clinical indices and gut microbiome data. The support vector machine (SVM) algorithm‐based model has the highest accuracy (AUC = 0.80). This model utilized a comprehensive feature set that combined clinical data (including sex, age, duration, platelet count, and bleeding scales) with selected microbial species (including *Bacteroides ovatus*, *Bacteroides xylanisolvens*, and *Parabacteroides gordonii*), alpha diversities, KEGG pathways, and microbial modules. This study will provide new ideas for the prediction of clinical CS efficacy, enabling informed decision‐making regarding the initiation of CS or personalized treatment in patients with ITP.

## Graphical abstract

This study uses gut microbiome data to predict corticosteroid response in patients with immune thrombocytopenia (ITP). Fecal samples from 212 patients with ITP are sequenced. Six machine‐learning algorithms are used to train predictive models. The support vector machine‐based model integrated clinical data and selected microbial species, diversities, and functional pathways, and has the highest accuracy.

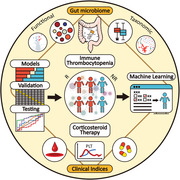

## 1 Introduction

Primary immune thrombocytopenia (ITP), an autoimmune hemorrhagic disorder, is characterized by isolated thrombocytopenia, with a peripheral blood platelet count of less than 100 × 10^9^/L in at least two routine blood tests and without abnormalities in blood cell morphology.^[^ ^1^ ^]^ Patients with newly diagnosed ITP (ND‐ITP) commonly receive corticosteroids (CSs) as initial treatment.^[^ ^2^ ^]^ However, more than 30% of patients with ITP respond poorly to CS therapy, and 50–85% of patients who respond to initial CS therapy relapse in the first year of treatment.^[^ ^3^ ^]^ To avoid CS‐related complications, prednisone should be rapidly tapered and usually discontinued in responders, especially non‐responders, after 4 weeks. A prolonged course of the disease and long‐term use of glucocorticoids, even at a low daily dose, such as 5 mg prednisone, can cause substantial toxicity and predispose patients to potentially life‐threatening bleeding events.^[^ ^3^ ^]^ Recently, better first‐line treatment options have been explored to achieve better initial and sustained remission and reduce bleeding events in patients with ITP, including combinations of drugs with other mechanisms (e.g., rituximab,^[^ ^4^ ^]^ all‐trans retinoic acid,^[^ ^5^ ^]^ and oseltamivir^[^ ^6^ ^]^) based on high‐dose dexamethasone or exploring new drugs.^[^ ^7^ ^]^ Therefore, the timely identification of patients likely to exhibit CS‐resistant ITP is critical, and new diagnostic methods are urgently needed to predict specific treatment responses.

ITP is a multifactorial disease with a complex pathogenesis.^[^ ^8^ ^]^ The pathogenesis of ITP may provide promising predictive characteristics to help differentiate patients with ITP who may respond to therapy from those who may develop resistance.^[^ ^9^ ^]^ During initial therapy, an abnormal CD4:CD8 ratio or CD8^+^CD25^str+^ Tregs may predict glucocorticoid response.^[^ ^10^ ^]^ Furthermore, a high C‐reactive protein level at diagnosis is linked to slow platelet count recovery three months after diagnosis.^[^ ^11^ ^]^ The following factors indicate a poor response to CS therapy for ITP: age, platelet count, platelet‐associated IgG, platelet distribution width, mean platelet volume, white blood cell (WBC) count or lymphocyte ratio, and megakaryocyte count in the bone marrow.^[^ ^12^ ^]^ However, there is still no method to predict a patient's course and response to treatment accurately.^[^ ^13^ ^]^

The use of the gut microbiota as a predictor of immunomodulatory therapy has increased in autoimmune and cancer immunotherapies.^[^ ^14^ ^]^ Individuals with rheumatoid arthritis may respond differently to treatment based on small gut, dental, or salivary microbiota changes.^[^ ^15^ ^]^ Certain gut microbiota, such as *Bifidobacterium* spp., may enhance the efficacy of anti‐programmed cell death 1 ligand 1 (PD‐L1) antibody therapy in melanoma.^[^ ^16^ ^]^ We previously observed dysbiosis in the phylogenetic composition and function of the ITP gut microbiome. We found that alterations in the gut microbiome in CS‐resistant ITP differed from those in treatment‐naïve ITP.^[^ ^17^ ^]^ Delving deeper into the gut microbiota to identify the species that can predict treatment outcomes or directly affect therapeutic efficacy may provide insightful guidance and innovative intervention strategies for the clinical management of ITP. Our study demonstrated that a machine learning model constructed from gut microbiome signatures can effectively identify patients with ITP who are at a heightened risk of developing CS resistance in the initial response assessment within the first four weeks of CS therapy. The efficacy of this model was confirmed through a rigorous multicenter validation study.

## 2 Results

### 2.1 Study Population

**Figure 1:**
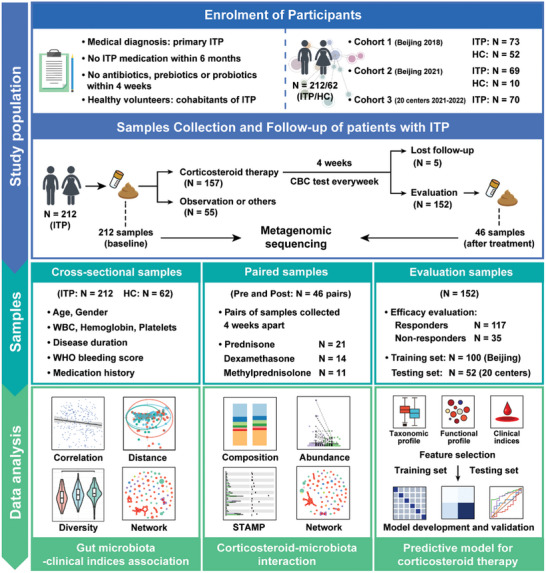
Flow diagram of study enrolment, sample collection, follow‐up, and data analysis. To evaluate the association between the gut microbiota and clinical indices and corticosteroid response, we profiled stool metagenomes of patients with primary ITP and healthy volunteers. Individuals were excluded if they were treated with any medication for thrombocytopenia in the previous 6 months; or exposed to antibiotics, prebiotics, or probiotics within 4 weeks before fecal sampling. In 2018 and 2021, individuals including patients with ITP (N = 142) and healthy volunteers (N = 62) were enrolled in a single center (cohort 1 and cohort 2) in Beijing, China. During 2021–2022, 70 participants with ITP were enrolled in the multicenter cohort (cohort 3). Patients with ITP subsequently received personalized treatment under clinical supervision, among which a total of 152 participants proceeded with standard corticosteroid therapy, completing an efficacy evaluation within four weeks post‐treatment initiation. Upon enrollment, all participants submitted baseline stool samples (ITP, N = 212; HC, N = 62), and 46 participants with ITP provided stool samples after the fourth week following the start of corticosteroid therapy. This study used all baseline sample data to conduct cross‐sectional descriptive studies and correlation analysis with population characteristics and clinical metrics. Key clinical indicators included the duration from initial ITP diagnosis at enrollment, WHO bleeding score, complete blood count parameters, and medication history over the previous six months. For the 46 paired samples before and after corticosteroid treatment, the interaction between corticosteroids and the gut microbiota was explored through microbial abundance and network analyses. Machine learning techniques were utilized for the 152 patients assessable for corticosteroid efficacy, merging baseline fecal microbiome data with clinical indicators to predict treatment outcomes. The training set included 100 samples from two cohorts in Beijing, while the testing set comprised 52 samples from the cohort across 20 centers. The predictive model, which integrates microbial taxonomy, functional components, and clinical indicators, offers valuable assistance in clinical treatment decisions for ITP. Abbreviations: ITP, immune thrombocytopenia; HC, healthy controls; CBC, complete blood count; WBC, white blood cells; WHO, World Health Organization; STAMP, statistical analysis of metagenomics profile.

Patients included in this study were individuals aged 14 or older with primary ITP who were characterized by isolated thrombocytopenia without any obvious initiating and/or underlying cause. Secondary ITP, such as those with thrombocytopenia caused by drugs, viral infections, and hematological malignancies, was ruled out. At enrolment, patients with ITP were preliminarily screened based on their medical history, requiring that they had neither received any medication for thrombocytopenia in the past six months nor any antibiotics, prebiotics, or probiotics within 4 weeks (**Figure** **1**). Two hundred and twelve patients with thrombocytopenia were enrolled in the period 2018−2022, and stool samples were provided as baseline samples. Sixty‐two healthy cohabitants of ITP patients contributed fecal samples to serve as healthy controls (HCs). The detailed clinical characteristics are presented in Table S1 (Supporting Information). At the subsequent follow‐up, 152 patients received CS treatment and completed an efficacy evaluation within four weeks following the start of corticosteroid therapy (prednisone, n = 21; methylprednisolone, n = 11; and high‐dose dexamethasone, n = 14). A response was defined as a platelet count increase of at least 30 × 10^9^/L on two separate occasions within four weeks post‐treatment initiation, with a minimum interval of 7 days between measurements and doubling of the baseline platelet count in the absence of bleeding. Ultimately, 117 patients met our primary response outcome. In addition to the baseline samples, 46 subjects with ITP provided fecal samples 4 weeks after the initiation of corticosteroid therapy for a self‐controlled study before and after treatment.

### 2.2 Gut Microbiota Profiles Associated with the Clinical Indices of ITP

Various host factors of the human body influence the gut microbiota. We enrolled 212 patients with primary ITP and 62 HCs in the baseline cross‐sectional analysis of the study to explore the association between gut microbiota and clinical indices (Table S2, Supporting Information). The median age of the ITP group was 48 years (interquartile range [IQR]: 32–58 years), which is higher than the 38 years of the HC group. The proportion of females in the ITP group (67%) was significantly higher than that in the HC group (45%). The median disease duration of patients with ITP was 12 months (ND‐ITP/persistent ITP, n = 108; chronic ITP, n = 104). Upon enrolment, the median platelet count for patients with ITP was 25.0 × 10^9^/L (IQR: 9.0, 45.0), with the minimum value recorded at 1 × 10^9^/L. More than half of the ITP patients surveyed (54%, n = 115) had received CS treatment over six months prior to the study, and they were designated as the Y‐CS group. In contrast, patients without any prior history of CS treatment were categorized into the N‐CS group. Sixty‐three percent of the patients described bleeding symptoms at the clinic visit, 49% were rated as grade 1 according to the World Health Organization (WHO) bleeding scale system, and only one patient was assessed as grade 4. Taking into account the wide age range and geographical differences among the subjects, we performed correlation analyses to determine whether these factors influence the composition of the gut microbiota in fecal samples. The canonical correlation analysis (CCA) revealed that age is significantly correlated with the composition of the gut microbiota (Figure S1a and Table S3, Supporting Information, *p*‐value = 0.007, importance = 0.0622). By incorporating age as a fixed effect, multivariate association with linear models (MaAsLin2) analysis allows for the control of age‐related confounding factors and has identified a negative correlation between subjects' platelet counts and the abundance of specific bacterial species such as *Clostridium clostridioforme*, *Dorea longicatena*, *Bifidobacterium longum*, *Riminococcus gnavus*, *Ruminococcus torques*, and *Clostridioides difficile*. Notably, after adjustment, *Lactobacillus mucosae* showed a positive correlation with age, with a coefficient of 1.25 and a false discovery rate (FDR) of 0.015 (Figure S1b and Table S4, Supporting Information).

**Figure 2:**
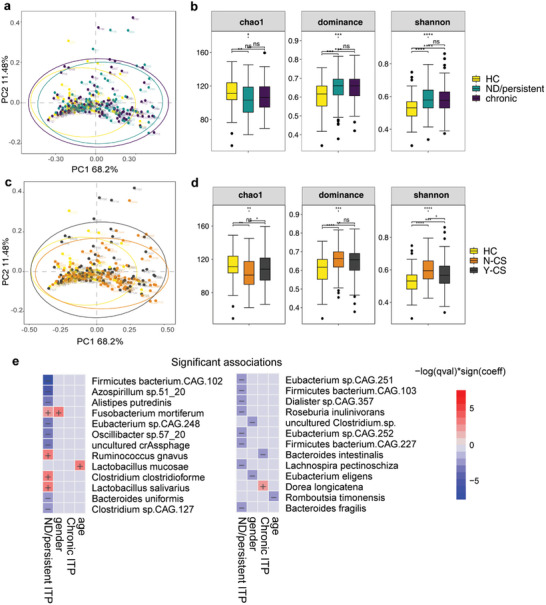
Gut microbiota profiles associated with the clinical indicators of ITP. a) The PCoA of β‐diversity based on order distribution by Bray‒Curtis dissimilarity in the HC group (n = 62), as well as the ND/persistent (n = 108) and chronic (n = 104) ITP groups. b) Comparison of the α‐diversity measured in the Chao1, Dominance, and Shannon index at the order level among the HC group (n = 62), as well as the ND/persistent (n = 108) and chronic (n = 104) ITP groups. Each box represents the IQR with the midpoint of the data. Whiskers indicate the upper and lower values within 1.5 times the IQR. c) The PCoA of β‐diversity based on order distribution by Bray‒Curtis dissimilarity in HC (n = 62), N‐CS (n = 97), and Y‐CS (n = 115) groups. d) Comparison of the α‐diversity measured with the Chao1, Dominance, and Shannon index at the order level among the HC (n = 62), N‐CS (n = 97), and Y‐CS (n = 115) ITP groups. e) Significant associations between gut microbial taxa and ITP grouping at the species level analyzed by MaAsLin2. *P*‐values are calculated using Kruskal–Wallis test, followed by the Dunn post‐hoc test across groups, ^*^ *p* < 0.05, ^**^ *p* < 0.01, ^***^ *p* < 0.001. Data from the figures was presented in Tables S5−S9 (Supporting Information). Abbreviations: CS, corticosteroid; ITP, immune thrombocytopenia; HC, healthy controls; PCoA, principal co‐ordinates analysis; LDA, Linear discriminant analysis; LEfSe, LDA Effect Size; IQR, interquartile range.

We proceeded to explore whether there were differences in gut microbiota composition among the various subgroups of ITP. Principal coordinate analysis (PCoA) based on the gut taxonomic profiles illustrated global differences between the HC, ND/persistent, and chronic ITP groups at the order level [permutational multivariate analysis of variance (PERMANOVA): *p* = 0.001] (**Figure** **2**a; Table S5, Supporting Information). Compared with HCs, patients with ND/persistent ITP had markedly lower alpha diversity according to the Chao1 and dominance indices, calculated based on the taxon order (Figure 2b; Table S6, Supporting Information). The PCoA results revealed global differences between HCs and patients with ITP in Y‐CS or N‐CS groups [PERMANOVA: *p* = 0.001] (Figure 2c; Table S7, Supporting Information). Patients with ITP in both Y‐CS and N‐CS groups had a higher Shannon index than that of the HC group, and significant differences were observed between the Y‐CS and N‐CS subgroups (Figure 2d; Table S8, Supporting Information). However, the chao1 index of the N‐CS group was significantly reduced compared to that of HC and Y‐CS groups. The three alpha diversity indices captured the variations in microbial richness and evenness observed in the fecal samples.

To confirm the features of gut microbiota profiles and their correlations with clinical indicators in ITP, we employed MaAsLin, controlling for age and gender as fixed effects, and included two classification indices: one for subgroups based on hormone medication history (HC, N‐CS, Y‐CS, with HC as the control) and another for subgroups based on disease progression (HC, ND/persistent ITP, chronic ITP, with HC as the control). The analysis revealed that only subgroups related to disease duration had significant associations with the fecal microbiota. Notably, *Ruminococcus gnavus*, *Clostridium clostridioforme*, and *Lactobacillus salivarius* were positively correlated with the ND/persistent ITP subgroup, whereas *Alistipes putredinis* showed a significant negative correlation with this subgroup (Figure 2e; Table S9, Supporting Information). From the microbiota co‐occurrence networks, two densely connected modules composed of various genera affiliated with the Firmicutes and Proteobacteria phyla were observed in both the ITP subgroups and healthy individuals (Figure S2, Supporting Information).

### 2.3 Gut Microbiota Variations throughout CS Therapy

**Figure 3:**
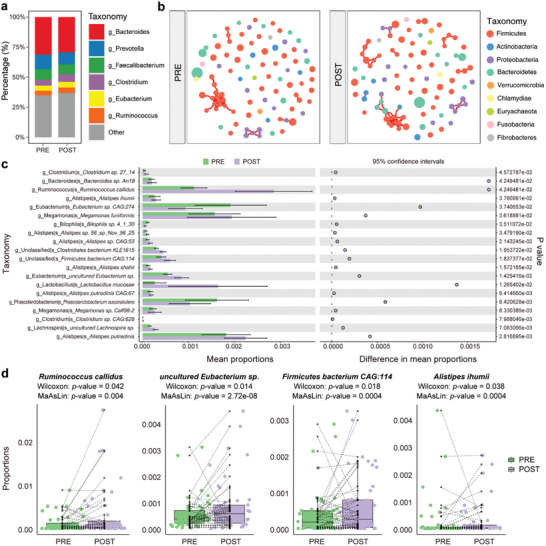
Gut microbiota variations throughout CS therapy. a) The relative abundance of predominant genera in the stool samples from patients with ITP (n = 46), collected both before (group PRE) and after (group POST) CS treatment. b) Gut microbial community networks constructed from genera‐level analysis of stool samples, from PRE and POST ITP groups that received CS treatment. A correlation coefficient >0.7 with statistically significant (*p* < 0.01) connections is shown (positive correlation, red edge; negative correlation, blue edge). Each node represents a genus, node size represents the relative abundance of the genus, and color represents the affiliated phylum. c) Relative abundance of the 20 species most associated with CS therapy between PRE and POST groups in STAMP analysis. *P*‐values are calculated using paired Wilcoxon test, ^*^ *p* < 0.05, ^**^ *p* < 0.01, ^***^ *p* < 0.001. d) The differential gut microbial species in the pre‐ and post‐CS treatment samples of ITP. PRE, n = 46; POST, n = 46. The four listed species were validated using both paired Wilcoxon and MaAsLin analysis. Each box represents the interquartile range (IQR, the range between the 25th and 75th percentiles) of the relative abundance with the mid‐point of the data. Dotted lines connect the points corresponding to samples collected before and after treatment from the same patient. (data was shown in Table S11, Supporting Information) Abbreviations: CS, corticosteroid; ITP, immune thrombocytopenia; STAMP, Statistical Analysis of Metagenomics Profile; MaAsLin, Multivariable Association with Linear Models.

To further investigate the impact of short‐term CS application on the gut microbiota, we collected samples from a prospective cohort of patients with ITP before and after treatment for a longitudinal comparative analysis. Forty‐six patients diagnosed with ITP were enrolled and treated with CS, with a follow‐up period of 4 weeks post‐treatment (Table S10, Supporting Information). After CS therapy, a response was achieved in 36/46 (78.3%) of patients within 4 weeks. The post‐CS samples demonstrated no significant alteration in microbial diversity compared with that of the pre‐CS samples (data not shown). At the genus level, the changes in the abundance of the six most abundant genera before and after treatment were not statistically significant. Among these, genus *Clostridium* showed an increasing trend, whereas *Bacteroides* and *Prevotella* decreased (**Figure** **3**a). However, in the microbial community networks, we observed that two genera from Bacteroidetes, one genus from Proteobacteria, and another genus from Actinobacteria were associated with the original Firmicutes‐dominated module and new complex colonies were born (Figure 3b). To screen for variations in the gut microbiota at the species level, paired Wilcoxon test and MaAsLin analysis were performed. There were 21 differentially abundant species between the pre‐ and post‐CS samples (Figure 3c; Table S11, Supporting Information). After adjusting for age and gender through MaAsLin analysis, four species still exhibited significant associations with treatment (Figure 3d). An increase in *Ruminococcus callidus*, which belongs to the *Ruminococcus* genus, was observed in the post‐CS subgroup (Figure 3d). Six species in the *Alistipes* genus, especially *Alistipes ihumii* (Figure 3c,d), markedly increased following CS treatment. Particularly, as species with high abundance in the gut, *Phascolarctobacterium succinatutens*, *Eubacterium sp. CAG:274* and *Bilophila sp. 4_1_30* were significantly decreased following CS treatment, while *Lactobacillus mucosae* and *Megamonas funiformis* were increased in abundance in the post‐CS group (Figure S3, Supporting Information). These findings suggest that CS therapy may affect the gut microbiota at the species level in patients with ITP.

### 2.4 Baseline Gut Microbiota Signatures Correlated with CS Response

Gut microbiota heterogeneity between ITP groups prompted us to investigate whether it determines the efficacy of CS therapy. Patients with ITP who underwent CS therapy—consisting of prednisone (n = 92), methylprednisolone (n = 36), and high‐dose dexamethasone (n = 24)—and completed follow‐up were categorized into CS responders (R group, n = 117) and non‐responders (NR group, n = 35) based on their response assessment at 4 weeks following treatment initiation. **Table** **1** summarizes the patients’ demographic characteristics. A training set consisting of samples from cohort 1 and 2 in Beijing (n = 100) was used for comparative feature analyses and model development, and the remaining samples from the multicenter cohort 3 (n = 52) were used as the testing set for subsequent model validation (Table S12, Supporting Information).

**Table 1:** Baseline characteristics of the responder cohort and non‐responder cohort.

	Total [n = 152]	Non‐responders [n = 35]	Responders [n = 117]	*P*‐value
Demographics				
Age (years), median [IQR]	46.000[32.000,58.000]	47.000[32.0,55.0]	45.000[32.0,59.0]	0.618 ^a)^
Sex (male/female), n	47/105	17/18	30/87	0.010 ^b)^
Clinical				
Duration (months), median [IQR]	12.0[1.2,44.0]	20.0[7.0,84.0]	12.0[1.0,36.0]	0.063 ^a)^
Type of ITP, n (%)				0.306 ^b)^
Newly diagnosed/persistent	81(53.289)	16(45.714)	65(55.556)	
Chronic	71(46.711)	19(54.286)	52(44.444)	
Bleeding symptoms, n (%)	99(65.132)	19(54.286)	80(68.376)	0.125 ^b)^
WHO bleeding scale, n (%)				0.170 ^b)^
Grade 0	53(34.868)	16(45.714)	37(31.624)	
Grade 1	73(48.026)	12(34.286)	61(52.137)	
Grade 2	26(17.105)	7(20.000)	19(16.239)	
Laboratory test				
WBC (×10^9^/L), median [IQR]	6.670[5.190,8.830]	6.400[4.290,9.270]	6.700[5.560,8.670]	0.458 ^a)^
HB (g/L), median [IQR]	133.0[121.0145.0]	135.0[113.0149.0]	133.0[123.0144.0]	0.941 ^a)^
PLT (×10^9^/L), median [IQR]	21.0[8.0,45.0]	35.0[18.0,52.0]	17.0[6.0,40.0]	0.006 ^a)^
Medication and intervention history				
Corticosteroid, n (%)	81(53.289)	24(68.571)	57(48.718)	0.039 ^b)^
IVIG, n (%)	8(5.263)	1(2.857)	7(5.983)	0.467 ^b)^
rhTPO / TPO‐RAs, n (%)	22(14.474)	6(17.143)	16(13.675)	0.609 ^b)^
Rituximab, n (%)	3(1.973)	1(2.857)	2(1.709)	0.668 ^b)^
Cyclosporine A, n (%)	15(9.868)	5(14.286)	10(8.547)	0.318 ^b)^
Danazol, n (%)	42(27.632)	12(34.286)	30(25.641)	0.316 ^b)^
Mycophenolate mofetil, n (%)	13(8.553)	4(11.429)	9(7.692)	0.488 ^b)^
Corticosteroid therapy				0.147 ^b)^
Prednisone, n (%)	92(60.526)	25(71.429)	67(57.265)	
Dexamethasone, n (%)	24(15.789)	2(5.714)	22(18.803)	
Methylprednisolone, n (%)	36(23.684)	8(22.857)	28(23.932)	

Data presented as median [interquartile range, IQR]. A two‐sided *p*‐value of less than 0.05 was established as the threshold for statistical significance. Abbreviations: ITP, immune thrombocytopenia; WHO, World Health Organization; WBC, white blood cells; HB, hemoglobin; PLT, platelet; IVIG, intravenous immunoglobulin; rhTPO, recombinant human thrombopoietin; TPO‐RA, thrombopoietin receptor agonists (e.g., Eltrombopag, Herombopag, Avatrombopag, etc.); IQR, interquartile range;

^a)^ Calculated using the Mann–Whitney U test;

^b)^ Calculated using the χ^2^ test.

**Figure 4:**
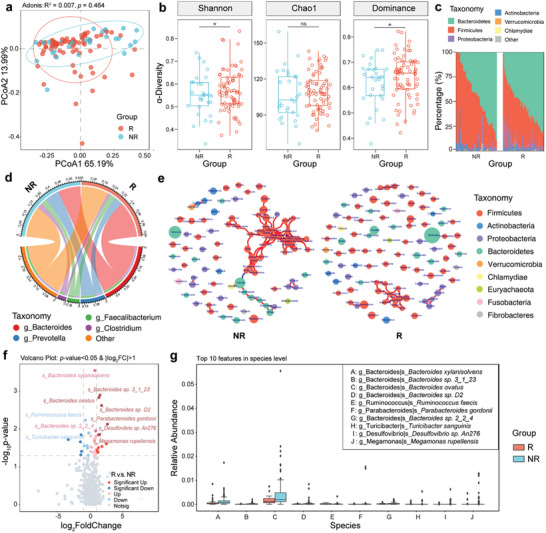
The baseline gut microbiota composition of patients with ITP was correlated with CS response. a) PCoA of β‐diversity based on order distribution by Bray‒Curtis distance between responders (group R, n = 71) and non‐responders (group NR, n = 29) to CS therapy. Adonis analysis was used to test the statistical significance of β dissimilarities between groups. b) Comparison of the α‐diversity measured with the Chao1, dominance, and Shannon indices at the order level between the R (n = 71) and NR (n = 29) groups. Each box represents the interquartile range (IQR, the range between the 25th and 75th percentiles) of the relative abundance with the mid‐point of the data. *P*‐values are calculated using Mann–Whitney U test, ^*^ *p* < 0.05, ^**^ *p* < 0.01, ^***^ *p* < 0.001. c) The overall composition and relative abundance of the bacterial community at the phylum level between the R (n = 71) and NR (n = 29) groups. d) Circos plot of the distribution of abundant genera in the R (n = 71) and NR (n = 29) groups. Groups are represented above, genera are represented below, chords connect different genera and samples, and the outer circle indicates the relative abundance of the genus. e) Gut microbial community networks based on genera in the R (n = 71) and NR (n = 29) groups, constructed by using Spearman correlations. A correlation coefficient >0.7 with statistically significant (*p* < 0.01) connections is shown (positive correlation, red edge; negative correlation, blue edge). Each node represents a genus, node size represents the relative abundance of the genus, and color represents the affiliated phylum. f) Volcano plot of the distribution of all differentially enriched species between the R (n = 71) and NR (n = 29) groups. g) Box plots of the top 10 species differentially enriched between the R (n = 71) and NR (n = 29) groups at baseline. Each box represents the IQR with the midpoint of the data. Whiskers indicate the upper and lower values within 1.5 times the IQR. *P*‐values are calculated using Mann–Whitney U test, ^*^ *p* < 0.05, ^**^ *p* < 0.01, ^***^ *p* < 0.001. Data from the figures was presented in Tables S13−S15 (Supporting Information). Abbreviations: ITP, immune thrombocytopenia; CS, corticosteroids; PCoA, principal coordinates analysis; R, responders; NR, non‐responders; IQR, interquartile range.

In the comparison between the R and NR groups, PCoA was unable to differentiate responses from non‐responses across taxonomic ranks (**Figure** **4**a; Table S13, Supporting Information). However, community diversity at baseline was significantly lower in patients who did not achieve a response at 4 weeks (Figure 4b). The NR group showed an increase in Firmicutes (F) and a decrease in Bacteroidetes (B) at the phylum level, suggesting a reduction in the B:F ratio (Figure 4c). At the genus level, Bacteroides showed a definite downward trend in NRs to CS therapy (Figure 4d). Within the baseline samples, differences in the response to CS were also evident in the microbiota networks (Figure 4e). The community dominated by Firmicutes was further expanded in the NR group and was negatively correlated with the microbial community formed by Bacteroidetes (including *Prevotella*, *Alistipes*, *Prevotellamassilia*, *Paraprevotella*, and *Butyricimonas*). LEfSe analysis identified a vastly increased tropism of the phylum Firmicutes in the R group and wide enrichment of the *Bacteroides* genus in the NR group (Figure S4 and Table S14, Supporting Information). As shown in the volcano plot, six species, including *Bacteroides ovatus*, *Bacteroides sp. 3_1_23*, *Bacteroides sp. D2*, and *Parabacteroides gordonii*, were significantly more abundant in the R group, while the abundances of *Ruminococcus faecis* and *Turicibacter sanguinis* were upregulated in the NR group (Figure 4f). Bacteroides accounted for half of the top ten species‐level enrichment features in the NR group (Figure 4g; Table S15, Supporting Information).

### 2.5 Functional Alterations in the Fecal Metagenome of ITP related to CS Treatment

**Figure 5:**
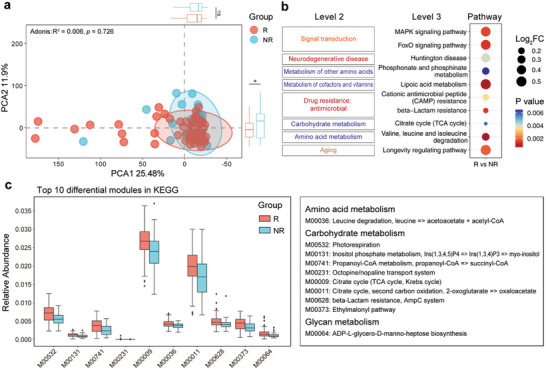
Functional alterations in the fecal metagenome of ITP were related to the CS response. a) Principal component analysis (PCA) of sample distribution based on KEGG gene annotation between responders (group R, n = 71) and non‐responders (group NR, n = 29) to CS therapy. Adonis analysis was used to test the statistical significance of β dissimilarities between groups. b) Dot plot of differentially annotated KEGG pathways. The dot size represents the log_2_FoldChange in the R group, and the color scheme represents *p* values. c) Box plots of the top 10 modules differentially enriched between the R (n = 71) and NR (n = 29) groups at baseline. Each box represents the IQR with the midpoint of the data. Whiskers indicate the upper and lower values within 1.5 times the IQR. *P*‐values are calculated using Mann–Whitney U test, ^*^ *p* < 0.05, ^**^ *p* < 0.01, ^***^ *p* < 0.001. Data from the figures was presented in Tables S16−S19 (Supporting Information). Abbreviations: ITP, immune thrombocytopenia; CS, corticosteroids; R, responders; NR, non‐responders; PCA, principal component analysis; KEGG, Kyoto Encyclopedia of Genes and Genomes; FC, fold change; IQR, interquartile range.

Functional metagenomic analysis was performed to determine whether the gut microbiota characteristics affected CS efficacy. After gene annotation based on the Kyoto Encyclopedia of Genes and Genomes (KEGG) database, principal component analysis (PCA) based on KEGG ortholog profiles revealed a significant difference in axis2 between the responders and non‐responders [Wilcoxon test on PCoA2: R versus NR, *p* = 0.033] (**Figure** **5**a; Table S16, Supporting Information). In contrast to the NR group, the R group exhibited several enriched KEGG pathways. The “lipoic acid metabolism,” “valine, leucine and isoleucine degradation” and “phosphonate and phosphonate metabolism” pathways (Level 3) elevated in the R group were involved in the “metabolism of cofactors and vitamins,” “amino acid metabolism,” and “metabolism of other amino acids” pathways (Level 2), respectively (Figure 5b; Table S17, Supporting Information). We observed that the abundances of the “Drug resistance: antimicrobial” and “Signal transduction” pathways (Level 2) were also increased in the R group (Figure 5b), which may be related to alterations in *Bacteroides*. Using MaAsLin, we delved deeper into the correlations between specific pathways and the effectiveness of CS treatment. With the adjustment for confounding variables including age and gender, the enrichment of the aforementioned pathways in the R group was reaffirmed. Beyond the influence of age and gender, a CS medication history of more than six months before study entry was identified as having a significant effect on the functional components of the fecal microbiota, notably leading to a reduction in “Tropane, piperidine, and pyridine alkaloid biosynthesis,” “Arginine and proline metabolism” “Tyrosine metabolism” and “Isoquinoline alkaloid biosynthesis” functions (Figure S5 and Table S18, Supporting Information). The Mann–Whitney U test was used to identify 10 significantly different modules between the R and NR groups based on the baseline samples (Figure 5c; Table S19, Supporting Information). Impressively, modules related to the “citrate cycle” were overrepresented in the R group, with a higher abundance. Functional analysis has revealed that gut dysbiosis interferes with physiological metabolic functions during disease and treatment.

### 2.6 Predictive Models for CS Response based on Taxonomic and Functional Components

**Figure 6:**
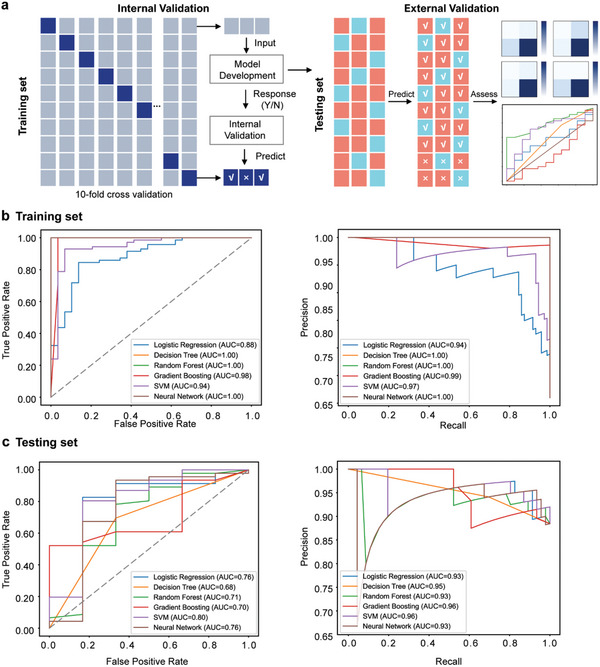
Predictive models for CS response in patients with ITP based on taxonomic and functional components. a) Machine learning framework for predicting CS response: training set, n = 100; testing set, n = 52. b−c) AUROC, PR AUC, and MCC values for CS response based on a combination of clinical indices, alpha diversities, taxonomic components, and functional components in the training b) and testing c) sets using six models. Abbreviations: ITP, immune thrombocytopenia; CS, corticosteroids; AUC, the area under the curve; SVM, support vector machines; AUROC, receiver operating characteristic curve; PR AUC, the precision‐recall area under the curve.

To test whether the gut microbiome can be used as a biomarker to predict the clinical response to CS treatment in patients with ITP, we aimed to derive and evaluate models of the gut microbial taxonomic and functional components at baseline to predict the clinical response using six machine learning models. Model development and internal validation were performed on the training set, and external validation was performed on the testing set. The area under the receiver operating characteristic curve (AUROC), the precision‐recall area under the curve (PR AUC), and the Matthews correlation coefficient (MCC) were obtained to compare the model performance (**Figure** **6**a). A subset of taxa that were differentially enriched between the R and NR groups was identified by preselecting the microbial features in the training set (Table S20, Supporting Information). While random forest (RF) and gradient boosting machine (GBM) performed well in the training set (AUROC = 1.00), their external validation results were less satisfactory, with AUROCs of 0.71 and 0.70, respectively. The support vector machine (SVM) model emerged as the top‐performing approach, achieving an AUROC of 0.80 and a PR AUC of 0.96 in the external validation set.

The mean AUROC of the SVM at the species level (0.77) was marginally lower than that of the SVM when all features were combined (0.80), underscoring the value of integrating diverse data types for better predictive accuracy (Table S20, Supporting Information). Considering the improved performance with species‐level features, these were used alongside taxonomic relative abundance, KEGG pathways, and diversity indices to ensure high‐resolution prediction.

Four combinations of predictors were tested to construct the models. Clinical baseline data alone were inadequate to predict the response at week 4, achieving an AUROC of 0.52, PR AUC of 0.90, and an MCC of 0.00 (Figure S6 and Table S21, Supporting Information). The inclusion of taxonomic features significantly improved the performance, with the species‐level SVM model attaining an AUROC of 0.77, PR AUC of 0.93, and an MCC of 0.43. Functional modules also contributed moderate enhancements (AUROC = 0.71; PR AUC = 0.92; MCC = 0.35), while diversity indices yielded limited predictive gains (AUROC = 0.54; PR AUC = 0.91; MCC = 0.03). The best‐performing model combined all data types (clinical, taxonomic, and functional) in the SVM (Figure 6b,c), resulting in the highest overall accuracy with an AUROC of 0.80, PR AUC of 0.96, and MCC of 0.37. This was superior to other classifiers such as RF (AUROC = 0.75; PR AUC = 0.93; MCC = 0.19) and neural network (NN, AUROC = 0.76; PR AUC = 0.93; MCC = 0.26). Detailed MCC values and selected thresholds across folds are summarized in Table S22 (Supporting Information).

In conclusion, the SVM model incorporating clinical, taxonomic, and functional data provided the highest classification accuracy. By evaluating performance metrics including PR AUC and MCC, alongside AUROC, our study delivers a comprehensive assessment of predictive models, addressing challenges associated with dataset imbalance. The resulting predictive model will be deployed online as a decision‐support application, allowing clinicians to leverage patient‐specific microbiome and clinical data for treatment planning (Figure S7, Supporting Information).

## 3 Discussion

Here, we established a new predictive model of CS response in ITP from the perspective of intestinal microbiota composition, which is different from previous models of immune cells or inflammatory substances. The development of omics technology and artificial intelligence algorithms in the last decade, such as machine learning, has led to the discovery of a variety of novel biomarkers for medical research. For example, in tumor research, machine learning is used to create and compare up to 128 predictive models.^[^ ^18^ ^]^ This helps avoid missing the best models and highlights other important biological traits. In pancreatic cancer studies, predictions based only on clinical data are the least accurate.^[^ ^18a^ ^]^ In breast cancer studies, incorporating miRNA isoform indicators has proven to predict the response of breast cancer patients to doxorubicin with greater accuracy than the traditional single‐gene marker HER2.^[^ ^18b^ ^]^ Machine learning has been previously utilized to build diagnostic models for ITP. Zuo et al.^[^ ^19^ ^]^ demonstrated that a stepwise logistic regression (LR) model, along with ROC curves, found positive diagnostic values for 10 plasma miRNAs that characterize patients with ITP within treatment responder and non‐responder groups. Based on our previous study, we used an RF model to establish a diagnostic model with 12 species, resulting in 92.29% accuracy for the training set and 83.61% accuracy for the test set, suggesting that information on the gut microbiota can be used to accurately identify patients with ITP.^[^ ^17^ ^]^ Moreover, patients with relapsed or steroid‐resistant ITP showed a unique gut microbiota composition. Therefore, we propose that the gut microbiota plays a role in the efficacy of ITP treatment.

In this study, we explored multiple predictive modeling approaches, ultimately identifying the SVM as the most effective method, achieving an AUROC of 0.80 in the external validation set. Drawing on the construction methods of gut microbiome prediction models in previous studies of intestinal or liver diseases,^[^ ^20^ ^]^ data were categorized into four primary modules—clinical data, taxonomic information, functional pathways (e.g., KEGG Level 3), and diversity indices—to assess their individual and combined contributions to prediction accuracy.^[^ ^21^ ^]^ Feature selection was conducted within each module to pinpoint the most pertinent variables. Additionally, grid‐search methods were employed to fine‐tune model parameters, thereby guaranteeing robust and reliable performance. The clinical data module alone demonstrated modest predictive power, with an AUROC of 0.70 in the external validation set.^[^ ^12b^ ^]^ However, when microbiome data were integrated, particularly taxonomic features, the predictive performance significantly improved. The combined use of clinical indicators and taxonomic relative abundance enhanced the SVM model's ability to distinguish responders from non‐responders, reflecting the value of incorporating microbiome data into predictive models. The enhanced performance of the SVM model highlights its capability to effectively handle complex, high‐dimensional data and underscores its potential as a reliable tool for guiding personalized treatment strategies in patients with ITP. By leveraging both clinical and microbiome data, this approach offers a promising avenue for improving treatment decision‐making and patient outcomes.

Meanwhile, the gut microbiota of patients with ITP treated with CS was further investigated, both cross‐sectionally and longitudinally, to offer a more detailed and thorough depiction of the alterations in the gut microbiota across various ITP subgroups, as well as the shifts that occur pre‐ and post‐treatment. At the phylum level, the proportion of Firmicutes and Bacteroidetes varies across different ITP studies. The reduced abundance of Bacteroidetes in fecal samples shows a clear correlation with age.^[^ ^22^ ^]^ *Alistipes*, which belong to the Bacteroidetes phylum, were significantly decreased in the fecal samples of ND/persistent ITP. *Alistipes ihumii* has been isolated from the feces of patients with anorexia nervosa^[^ ^23^ ^]^ and is mainly an increased taxon before and after CS treatment. The abundance of *Alistipes* was negatively correlated with the Firmicutes community in the NR group at baseline. *Alistipes*, an anaerobic symbiotic bacterium identified in clinical medical samples, is bile resistant^[^ ^24^ ^]^ and can produce short‐chain fatty acids as catabolism metabolites.^[^ ^25^ ^]^ This genus comprises 13 species, including *A. finegoldii*, *A. putredinis*,^[^ ^26^ ^]^ *A. shahii*,^[^ ^27^ ^]^ and *A. ihumii*.^[^ ^23^ ^]^ *Alistipes* spp. are considered to have protective effects against liver diseases (e.g., liver cirrhosis^[^ ^28^ ^]^ and nonalcoholic fatty liver disease^[^ ^25^ ^]^), cardiovascular diseases (e.g., hypertension^[^ ^29^ ^]^ and atrial fibrillation^[^ ^30^ ^]^), and inflammatory bowel disease.^[^ ^31^ ^]^ *Alistipes* is predominantly recognized as a consequence of treatment responses. Undoubtedly, future proactive interventions could reveal its potential role in CS therapy for patients with ITP. In the final predictive model for CS response, some informative probiotics, such as *Bacteroides ovatus*, *Bacteroides xylanisolvens*, and *Parabacteroides gordonii*,^[^ ^32^ ^]^ with high abundance were included, and all were enriched in the R group. *Parabacteroides* spp. have been reported to be beneficial for weight loss in patients with obesity.^[^ ^33^ ^]^ *Bacteroides ovatus* plays a role in regulating intestinal immunity,^[^ ^34^ ^]^ and *Bacteroides xylanisolvens* has been demonstrated to increase the content of folic acid in the liver and blood, which is beneficial in lowering lipid and glucose levels and conferring an anti‐fatty liver effect.^[^ ^35^ ^]^ *Ruminococcus faecis*,^[^ ^36^ ^]^ which was enriched in the NR group, belongs to the *Ruminococcus* genus, is similar to the *Ruminococcus gnavus* isolate we identified in newly diagnosed ITP,^[^ ^17^ ^]^ and may be associated with inflammatory responses.^[^ ^37^ ^]^ However, an increased presence of *Ruminococcus callidus* has been observed in the feces of patients with ITP after corticosteroid treatment. This bacterium plays a protective role in cancer immunotherapy and is crucial for the degradation of resistant starch.^[^ ^38^ ^]^ Its abundance is notably reduced in patients with inflammatory bowel disease and Crohn's disease.^[^ ^39^ ^]^ This suggests that different species within the same genus may have distinct functions. Further research is needed to better understand how gut microbiota can be heterogeneous and contribute to therapeutic resistance.

The study of gut microbiota currently necessitates further development and refinement in both methodology and underlying mechanisms. As the gut microbiota is affected by individuals, the environment, and collection and detection technologies, its application has been controversial. Microbial co‐occurrence network analysis is a powerful tool that continues to evolve, offering new opportunities to explore the complex world of microbiomes and their intricate relationship with human health and disease. Age is a confounding factor that cannot be ignored in the current and subsequent studies. To overcome the influence of region and diet on gut microbiota, we used multicenter samples from different regions, ethnic groups, and dietary habits as external validation data to test the validity of the model. As fully automated machine learning platforms continue to evolve, omics data will increasingly furnish a greater array of clinical models and enhance the information available for decision‐making processes.^[^ ^40^ ^]^ However, we acknowledge that a more thorough and extensive validation in larger cohorts is crucial before the model can be applied in clinical practice. Considering the practicality and generalization of the model, the detection technology of the gut microbiota needs to be further refined and simplified, such as the calculation of absolute abundance and unification of different sequencing technologies, which are the focus of future implementation. Developing effective models to identify and guide medication administration in patients with ITP who are resistant to first‐line therapy remains challenging.

## 4 Conclusion

This study innovatively constructed machine learning models based on clinical factors combined with gut microbiota biomarkers, which improved the prediction efficiency via noninvasive detection to guide personalized therapies for patients with ITP.

## 5 Experimental Section

### Study Participants

The inclusion criteria were ages ≥14 years and a diagnosis of ITP based on the internationally accepted criteria for primary ITP.^[^ ^3^ ^]^ At initial diagnosis, a complete history (including bleeding after previous surgery, dentistry, or trauma, prior blood counts, drug and toxin exposure, recent foreign travel and vaccinations, recent infections, needle‐stick accidents, and prior transfusions with blood products), physical examination, blood count, and peripheral blood film analyses were performed. Detection of *Helicobacter pylori*, hepatitis B virus (HBV), human immunodeficiency virus (HIV), and hepatitis C virus (HCV) infections; blood antibodies against nuclei; antiphospholipid antibodies; antithyroid antibodies; and thyroid hormone levels was performed in adult patients with suspected ITP. The exclusion criteria were as follows: secondary ITP, such as drug‐associated thrombocytopenia; thrombocytopenia caused by viral infection (HIV, HBV, or HCV); myelodysplastic disorder or myelofibrosis; nursing or pregnant women; severe dysfunction of the heart, kidney, lung, or liver; and active or previous malignancy. Patients were excluded if they were treated with any medication for thrombocytopenia in the previous 6 months; exposed to antibiotics, prebiotics, or probiotics within 4 weeks; or treated with immunosuppressants before fecal sampling. Patient records of ITP treatment administered more than 6 months prior were documented within their medication and intervention history. Based on the clinical course of ITP, the patients enrolled in this study were classified as having newly diagnosed (< 3 months), persistent (3–12 months), or chronic (> 12 months) ITP.^[^ ^41^ ^]^ For the evaluation of bleeding manifestations, the WHO scale (Grade 0–5) was used to identify patients with ITP at risk of bleeding in this study.^[^ ^42^ ^]^ The physician graded the patient at the time of the visit based on a physical examination or the patient's history supplemented with available medical records.

### Sample Collection and Follow‐up

This study consisted of three cohorts. All consecutive patients who met the inclusion criteria in the outpatient clinic at Peking University People's Hospital (Beijing, China) between March and September 2018 were included in cohort 1 (n = 73). Between April and November 2021, all patients who met the inclusion criteria at Peking University People's Hospital (Beijing, China) were consecutively recruited for this study (n = 69). The above two practices of single‐center enrolment were conducted following the same standardized operating procedures to ensure consistency in participant management and data collection. Sixty‐two healthy cohabitants of patients with ITP contributed fecal samples to serve as healthy controls. Each participant underwent identical clinical examinations and sampling protocols, including stool sample collection using *Invitek* Stool Collection Tubes with DNA Stabilizer. Between October 2021 and January 2022, seventy consecutive patients who met the inclusion criteria from 20 medical centers across China were enrolled in cohort 3. All participants provided stool samples for deep shotgun metagenomic sequencing at enrolment (Table S23, Supporting Information). The subjects voluntarily provided fecal samples 4 weeks after the initiation of corticosteroid therapy, and ultimately, 46 post‐treatment fecal samples paired with pre‐treatment samples were obtained. The Declaration of Helsinki guidelines were followed when conducting this study. All participants provided written informed consent before sampling and the study was approved by the Peking University People's Hospital Ethics Committee. The study was registered at ClinicalTrials.gov (NCT05118126).

### CS Therapy and Outcome

In this study, 152 patients received CS treatment during subsequent follow‐up. According to the international consensus on the treatment of primary ITP, the patients in this study were treated with prednisone (n = 92), methylprednisolone (n = 36), or high‐dose dexamethasone (n = 24).^[^ ^3^ ^]^ Prednisone was orally administered at a dose of 1 mg kg^−1^ for two weeks and for a maximum of three weeks. Methylprednisolone was also orally administered at a dosage equivalent to that of prednisone. If there was no response to the initial dose within two weeks, prednisone and methylprednisolone were tapered rapidly over one week and then stopped. Dexamethasone was administered orally or intravenously at a dose of 40 mg daily for four days and then stopped. If the patient continued to have platelets < 30 × 10^9^/L or had bleeding symptoms by day 10, an additional 4‐day course of dexamethasone (40 mg daily) was given.^[^ ^43^ ^]^ The platelet count should be checked on two separate occasions, at least seven days apart, to assess the treatment response within four weeks post‐treatment initiation. A response was defined as an increased platelet count (≥30 × 10^9^/L), and the platelet count had to increase by at least two‐fold over the baseline platelet count without bleeding.^[^ ^1a^ ^]^ A platelet count of <30 × 10^9^/L, an increase of < twofold over the baseline platelet count, or bleeding was deemed as no response.^[^ ^3^ ^]^ Subsequently, one hundred‐seventeen patients responded to the corticosteroid therapy, while thirty‐five patients did not respond within four weeks.

### Data Preprocessing

The integrated dataset was segmented (Table S24, Supporting Information) into four primary domains: microbiota taxonomy, diversity, functional attributes, and clinical parameters. The microbiota data encompassed multiple taxonomic levels, including phylum, class, order, family, genus, and species, with the top 10 differential taxa datasets from the Mann–Whitney test for each level (filtered by mean relative abundance >0.01%). Moreover, to characterize the microbial diversity in the samples, the diversity of the microbiota at the respective taxonomic levels assessed by the Chao1, dominance, and Shannon indices was used for model development. For functional components, datasets based on KEGG modules were used for modeling. These components were screened in the same way as the taxa and the final top 10 differential components were included for modeling. The clinical indicators included sex, age, duration, platelet count, bleeding scale score, and history of CS treatment.

### MaAsLin

MaAsLin models^[^ ^44^ ^]^ were used for the analysis of age, ITP subgroups, and KEGG functional pathways. The MaAsLin2 framework was imployed to adjust for age and other potential confounders such as gender (with the female as the reference), WBC, hemoglobin (HB), and geographical location. By including age as a fixed effect and participant ID as a random effect, MaAsLin2 controls for age‐related confounding and identifies significant taxa associated with the outcome of interest. To uncover microbial species linked to ITP subgroup disparities, the classification index including subgroups related to the history of corticosteroid medication (HC, N‐CS, Y‐CS, with HC as the reference), subgroups related to the course of the disease (HC, ND/persistent ITP, chronic ITP, with HC as the reference) and gender, as well as age were enforced. To explore the longitudinal changes in gut microbiota before and after CS treatment in patients with ITP, a MaAsLin analysis was conducted, with age and gender serving as fixed effects and patient ID as a random effect. Ultimately, MaAsLin was employed to scrutinize the correlations between microbial functional pathways and responses to CS therapy, with an emphasis on controlling for variables including treatment response (R and NR, with NR as the reference), age, gender, and prior corticosteroid use in the analysis.

### Machine Learning and Model Development

To develop a model for predicting the efficacy of CS therapy, patients who were lost to follow‐up and did not take drugs were excluded, and 152 patients who received CS treatment at a subsequent follow‐up were divided into training (cohort 1 and cohort 2, n = 100) and testing (cohort 3, n = 52) sets. Analysis of the microbiota data and therapeutic effectiveness of CS treatment in patients with ITP was conducted using machine learning models. The models were constructed using six machine‐learning algorithms: LR, decision tree (DT), RF, SVM, GBM, and NN.^[^ ^45^ ^]^ The feature selection was based on their statistical validity and potential predictive power across all four domains. Feature extraction techniques were deployed to transform high‐dimensional data into a lower‐dimensional form while retaining the essence of the original data. The training dataset was used for model construction and internal validation. The models were trained using a training dataset with hyperparameters optimized via grid search and cross‐validation techniques. Using the training set as an internal validation set, k‐fold cross‐validation was performed to assess the robustness and reliability of the model.^[^ ^46^ ^]^

### Model Evaluation and External Validation

Model performance was assessed using multiple metrics: AUROC, PR AUC, MCC, sensitivity, specificity, precision, recall, and F1 score, focusing on the AUROC, PR AUC, and MCC for imbalanced data. The PR AUC was calculated to provide an unbiased assessment of the precision and recall of the model under class imbalance. The MCC was chosen to further validate the performance of the classifier because it considers all confusion matrix elements and is robust for imbalanced datasets.

Models were finally tested on the independent external validation set (“test” type samples) to evaluate their predictive accuracy and generalizability to unseen data. This external validation helped to assess the real‐world applicability of the developed models. The performance of the models developed using different feature combinations from the four domains was compared. Ensemble methods were applied to combine the strengths of individual models and improve the overall predictive performance. The optimal decision threshold for each model was determined by maximizing the MCC during 5‐fold cross‐validation on the training cohort. Specifically, thresholds ranging were evaluated from 0.10 to 0.75 (increments of 0.05), and for each fold, the threshold was identified that achieved the highest MCC. The five best thresholds were then averaged from all folds to obtain the final cutoff. This final cutoff was subsequently applied to the independent testing cohort.

### Statistical Analysis

All participants included in the analysis provided complete data on the essential predictors (age, sex, platelet count, and other relevant clinical variables used in the model). White blood cell counts and hemoglobin data were missing for 14 patients because only critical platelet count changes were recorded in the medical records. The RF imputation was used to impute the remaining missing predictor values. Continuous variables were presented as median [IQR]. For continuous data, the Mann–Whitney U test was employed to analyze data features between two groups. When comparing more than two groups, the Kruskal–Wallis test was initially conducted, followed by the Dunn post‐hoc test to further examine differences across groups. Pearson's chi‐square test was utilized for categorical data analysis. A two‐sided p‐value of less than 0.05 was established as the threshold for statistical significance. PERMANOVA (also called Adonis analysis) was used to test the statistical significance of beta dissimilarities between selected groups. To compare the paired samples before and after CS therapy, the Wilcoxon paired test was used. Analyses were conducted using R software (version 4.3.1). For imputation, the mice package was employed; for PERMANOVA, the vegan package was used. Additionally, for pairwise comparisons, the stats package and ggplot2 packages were used for data visualization.

### Patient consent and ethics approval statement

All subjects provided written informed consent before sampling, and the Peking University People's Hospital Ethics Committee approved the study (No. 2021PHB437).

### Clinical trial registration

The study was registered on ClinicalTrials.gov (NCT05118126).

## Conflict of Interest

The authors declare no conflict of interest.

## Author Contributions

F.Q.L., Z.Y.A., L.J.C., and M.Y.X. contributed equally to this work. F.Q.L. and X.H.Z. performed in conceptualization. L.J.C., M.Y.X., Y.J.W., L.L.W., M.T.Z., and J.Y.Z. performed in data curation. Z.Y.A., Q.S.H., and F.Q.L. performed in formal analysis. F.Q.L., X.J.H., and X.H.Z. performed in funding acquisition. W.L., B.S.Z., L.Y., J.F., Z.G.L., R.F., Z.X.J., R.B.H., H.M.J., J.H.R., X.Y.Z., Y.F.C., Y.H.L., H.B.Z., D.G., Y.L., F.Y., X.W., J.L.Q., and D.H.H. performed in investigation. Z.Y.A., Q.S.H., and F.Q.L. performed in methodology. L.J.C. and X.H.Z. performed in project administration. Q.Y.Q., Q.C., Y.X.C., and M.L.L. performed in resources. Z.Y.A. performed in software. Q.F.W., Y.Y.L., and X.J.H. performed in supervision. H.X.F., F.Q.L., and Z.Y.A. performed in validation. F.Q.L. and M.Y.X. performed in visualization. F.Q.L. and Z.Y.A. performed in writing – original draft. L.J.C., X.J.H., and X.H.Z. performed in writing – review and editing.

## Supporting information



Supporting Information

Supplemental Tables

## Data Availability

The data that support the findings of this study are openly available in the Genome Sequence Archive in National Genomics Data Center, China National Center for Bioinformation / Beijing Institute of Genomics, Chinese Academy of Sciences (CRA016022)https://ngdc.cncb.ac.cn/gsa/s/sKC6u481.
