# Supplementary material for: Correlation Between Fecal Microbiota and Corticosteroid Responsiveness in Primary Immune Thrombocytopenia: an Exploratory Study
Source: Adv Sci (Weinh). 2025 Mar 5;12(22):2410417. doi: 10.1002/advs.202410417 (PMC12165022; doi:10.1002/advs.202410417)
Supplement: Supplementary file 1 — Supporting Information [file ADVS-12-2410417-s002.docx]

Supporting Information

Correlation between Fecal Microbiota and Corticosteroid Responsiveness in Primary Immune Thrombocytopenia: An Exploratory Study

Feng-qi Liu†, Zhuo-yu An†, Li-juan Cui†, Meng-yu Xiao†, Ye-jun Wu, Wei Li, Bang-shuo Zhang, Li Yu, Jia Feng, Zhuo-gang Liu, Ru Feng, Zhong-xing Jiang, Rui-bin Huang, Hong-mei Jing, Jin-hai Ren, Xiao-yu Zhu, Yun-feng Cheng, Yu-hua Li, He-bing Zhou, Da Gao, Yi Liu, Fan Yu, Xin Wang, Jian-lin Qiao, Dai-hong Hu, Lu-lu Wang, Meng-tong Zang, Qi Chen, Qing-yuan Qu, Jian-ying Zhou, Meng-lin Li, Yu-xiu Chen, Qiu-sha Huang, Hai-xia Fu, Yue-ying Li, Qian-fei Wang, Xiao-jun Huang and Xiao-hui Zhang*, on behalf of the Cooperative ITP Working Group

**Supplemental Methods**

**Fecal sampling and DNA extraction**

The Fecal samples were collected using standardized containers with a DNA stabilizer, which could be stored at room temperature for 3 months. Fecal samples were then transferred to the laboratory for further DNA extraction. According to the manufacturer's instructions, DNA was extracted from each sample (200 mg) using the QIAamp DNA Stool Mini Kit (Qiagen, Germany). The degree of DNA degradation and potential contamination were monitored on 1% agarose gels. The DNA concentration was measured using a Qubit® dsDNA Assay Kit in a Qubit® 2.0 Fluorometer (Life Technologies, CA, USA). The OD value was between 1.8 and 2.0, and DNA contents above 1 µg were used to construct the library.

**DNA library construction and sequencing**

A total of 1 μg DNA per sample was used as input material for the DNA sample preparations. Sequencing libraries were generated using the NEBNext® Ultra™ DNA Library Prep Kit for Illumina (NEB, USA) following the manufacturer’s recommendations, and index codes were added to attribute sequences to each sample. Briefly, the DNA sample was fragmented with sonication to a size of 350 bp, and then DNA fragments were end-polished, A-tailed, and ligated with the full-length adaptor for Illumina sequencing with further polymerase chain reaction (PCR) amplification. Finally, PCR products were purified (AMPure XP system), and libraries were analyzed for size distribution with an Agilent2100 Bioanalyzer and quantified using real-time PCR. The clustering of the index-coded samples was performed on a cBot Cluster Generation System according to the manufacturer’s instructions. After cluster generation, the library preparations were sequenced on an Illumina HiSeq platform, and paired-end reads were generated.

**Sequence processing and metagenome assembly**

Preprocessing the raw data (542,686.2 Mbp) obtained from the Illumina HiSeq sequencing platform using Readfq (V8, https://github.com/cjfields/readfq) was conducted to acquire the clean data for subsequent analysis. Clean data (541,352.6 Mbp) were then blasted to the host database by default using Bowtie2.2.4 software (Bowtie2.2.4, http://bowtiebio.sourceforge.net/Bowtie2/index.shtml) to filter the reads that were of host origin. The parameters ^[1]^ were as follows: --end-to-end, --sensitive, -I 200, and -X 400. Next, no host data (539,545.66 Mbp) were assembled and analyzed ^[2]^ with SOAPdenovo software (V2.04, http://soap.genomics.org.cn/soapdenovo.html), and the parameters ^[3]^ were as follows: -d 1, -M 3, -R, -u, -F, and -K 55. All samples’ clean data were compared to each scaffold with Bowtie2.2.4 software to acquire the PE reads not used, and the parameters were --end-to-end, --sensitive, -I 200, and -X 400. All the reads not used in the forward step of all samples were combined, and then SOAPdenovo (V2.04)/MEGAHIT (v1.0.4-beta) software was used for mixed assembly with the same parameters as a single assembly. The mixed-assembled scaffolds were broken from the N connection, and scaftigs were obtained. Fragments generated from single and mixed assemblies shorter than 500 bp in all scaftigs were filtered for statistical analysis.

Fecal metagenomic sequencing was performed using 274 stool samples (212 ITP, 62 HC), and a total of 2,830,699 gene sequences were obtained, with an average length of 734.79 bp. After taxonomic annotation, 133 phyla, 102 classes, 207 orders, 445 families, 1782 genera, and 9193 species were uniquely assigned, with a bacterial percentage of 91.03% at the kingdom level. Most bacteria belong to the following five phyla: Firmicutes, Bacteroidetes, Proteobacteria, Actinobacteria, and Fusobacteria.

**Bioinformatic analysis**

Fecal metagenomic sequencing was performed using 274 stool samples (212 ITP, 62 HC), and a total of 2,830,699 gene sequences were obtained, with an average length of 734.79 bp. After taxonomic annotation, 133 phyla, 102 classes, 207 orders, 445 families, 1782 genera, and 9193 species were uniquely assigned, with a bacterial percentage of 91.03% at the kingdom level. Gene prediction and abundance analysis, taxonomy prediction, and Kyoto Encyclopedia of Genes and Genomes (KEGG) functional database annotations were performed as previously described ^[4]^. USEARCH was used to calculate the sample α diversities (Chao1, dominance and Shannon index) from taxonomic relative abundance without filtering ^[5]^. Principal component analysis (PCA) ^[6]^(R ade4 package, Version 2.15.3) and principal coordinates analysis (PCoA) ^[7]^ (R vegan package, Version 2.15.3) decrease-dimension analysis were performed using binary Bray‒Curtis dissimilarities for β-diversity based on the relative abundance of each taxonomic hierarchy. This study mainly showed the α- and β-diversities of taxonomic and functional pathway abundances at the order and ko (KEGG genes) levels. The difference between groups was tested by Adonis analysis (R vegan package, Version 2.15.3). In taxonomic comparison analysis, taxa were prefiltered to be included with average relative abundances of >0.01% and then analyzed by the Mann–Whitney or paired Wilcoxon test provided by the Statistical Analysis of Metagenomics Profile (STAMP, v2.1.3). The linear discriminant analysis (LDA) effect size (LEfSe) algorithm was used to compare the abundance of taxa between the groups ^[8]^. LEfSe analysis was conducted with LEfSe software (the default LDA score was 2) ^[9]^. Canonical correspondence analysis (CCA) was performed (R rdacca.hp package 1.1-0) to obtain the p-value of each clinical characteristic after correlation analysis between the clinical indices and the composition of the gut microbiota ^[10]^. Microbial community networks were constructed using Spearman correlations and visualized in R software, with a threshold of correlation coefficient >0.7 and a statistically significant p-value of <0.01 ^[11]^.

**Supplemental References**

[1] a)F. H. Karlsson, V. Tremaroli, I. Nookaew, G. Bergstrom, C. J. Behre, B. Fagerberg, J. Nielsen, F. Backhed, *Nature* **2013**, 498, 99; b)F. H. Karlsson, F. Fak, I. Nookaew, V. Tremaroli, B. Fagerberg, D. Petranovic, F. Backhed, J. Nielsen, *Nat Commun* **2012**, 3, 1245.

[2] R. Luo, B. Liu, Y. Xie, Z. Li, W. Huang, J. Yuan, G. He, Y. Chen, Q. Pan, Y. Liu, J. Tang, G. Wu, H. Zhang, Y. Shi, Y. Liu, C. Yu, B. Wang, Y. Lu, C. Han, D. W. Cheung, S. M. Yiu, S. Peng, Z. Xiaoqian, G. Liu, X. Liao, Y. Li, H. Yang, J. Wang, T. W. Lam, J. Wang, *Gigascience* **2012**, 1, 18.

[3] a)N. Qin, F. Yang, A. Li, E. Prifti, Y. Chen, L. Shao, J. Guo, E. Le Chatelier, J. Yao, L. Wu, J. Zhou, S. Ni, L. Liu, N. Pons, J. M. Batto, S. P. Kennedy, P. Leonard, C. Yuan, W. Ding, Y. Chen, X. Hu, B. Zheng, G. Qian, W. Xu, S. D. Ehrlich, S. Zheng, L. Li, *Nature* **2014**, 513, 59; b)Q. Feng, S. Liang, H. Jia, A. Stadlmayr, L. Tang, Z. Lan, D. Zhang, H. Xia, X. Xu, Z. Jie, L. Su, X. Li, X. Li, J. Li, L. Xiao, U. Huber-Schonauer, D. Niederseer, X. Xu, J. Y. Al-Aama, H. Yang, J. Wang, K. Kristiansen, M. Arumugam, H. Tilg, C. Datz, J. Wang, *Nat Commun* **2015**, 6, 6528; c)J. U. Scher, A. Sczesnak, R. S. Longman, N. Segata, C. Ubeda, C. Bielski, T. Rostron, V. Cerundolo, E. G. Pamer, S. B. Abramson, C. Huttenhower, D. R. Littman, *Elife* **2013**, 2, e01202.

[4] Y. Wang, F. Liu, G. Zhang, Y. Su, X. Sun, Q. Chen, C. Wang, H. Fu, Y. He, X. Zhu, X. Liu, M. Lv, X. Zhao, X. Zhao, Y. Li, Q. Wang, X. Huang, X. Zhang, *Sci China Life Sci* **2021**, 64, 766.

[5] T. Rognes, T. Flouri, B. Nichols, C. Quince, F. Mahe, *PeerJ* **2016**, 4, e2584.

[6] E. Avershina, T. Frisli, K. Rudi, *Microbes Environ* **2013**, 28, 211.

[7] M. Noval Rivas, O. T. Burton, P. Wise, Y. Q. Zhang, S. A. Hobson, M. Garcia Lloret, C. Chehoud, J. Kuczynski, T. DeSantis, J. Warrington, E. R. Hyde, J. F. Petrosino, G. K. Gerber, L. Bry, H. C. Oettgen, S. K. Mazmanian, T. A. Chatila, *J Allergy Clin Immunol* **2013**, 131, 201.

[8] N. Segata, J. Izard, L. Waldron, D. Gevers, L. Miropolsky, W. S. Garrett, C. Huttenhower, *Genome Biol* **2011**, 12, R60.

[9] H. Yun, L. Sun, Q. Wu, G. Zong, Q. Qi, H. Li, H. Zheng, R. Zeng, L. Liang, X. Lin, *PLoS Med* **2020**, 17, e1003451.

[10] J. Lai, Y. Zou, J. Zhang, P. Peres-Neto, *bioRxiv* **2021**, 2021.03.09.434308.

[11] A. Limeta, B. Ji, M. Levin, F. Gatto, J. Nielsen, *JCI Insight* **2020**, 5.

**Supplemental Figures**


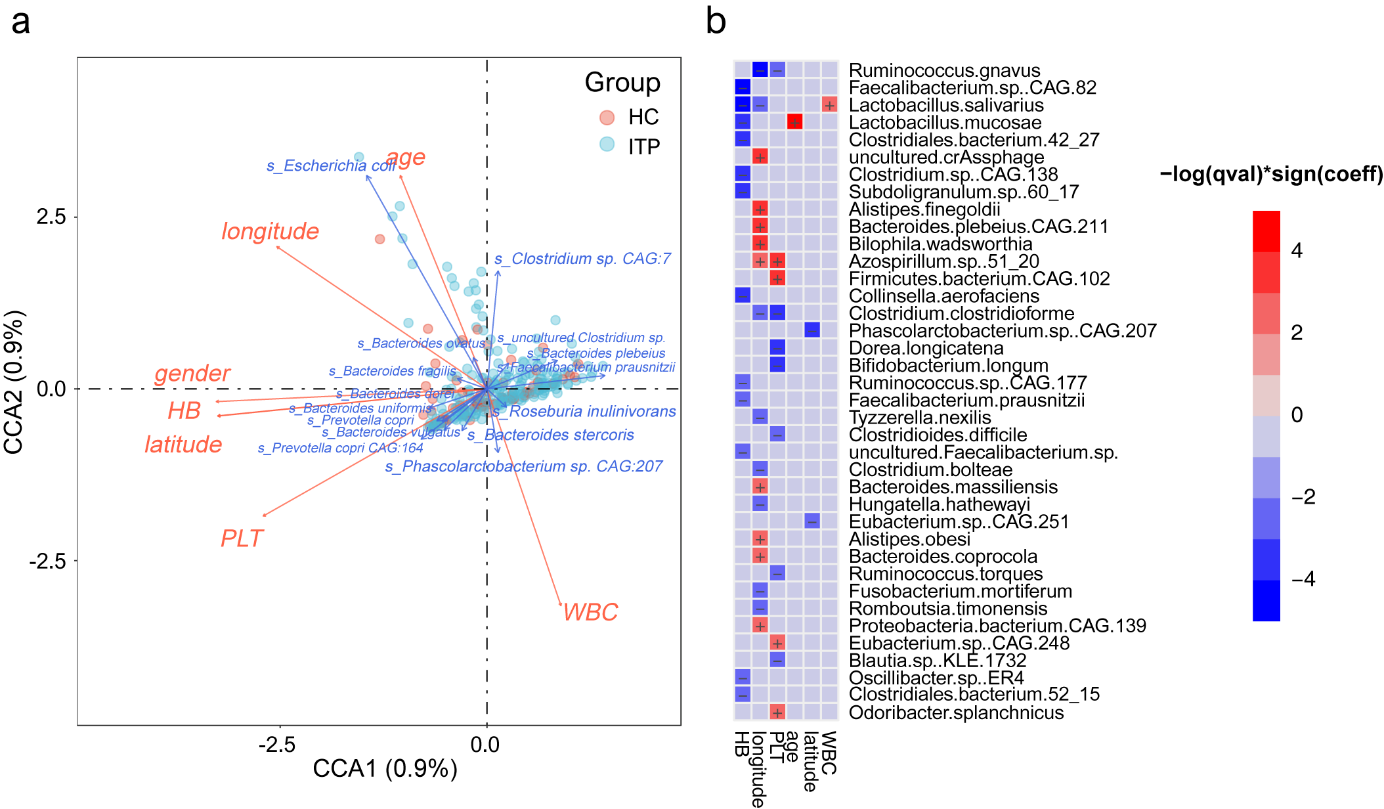


**Figure S1. Correlation analysis of the gut bacterial species and clinical indicators in the overall population.** a) Biplot of the clinical variables and the relative abundance of the top 15 species analyzed by CCA (n=274). The angle between species and clinical factors represented the positive and negative correlations (acute angle: positive correlation; obtuse angle: negative correlation; right angle: no correlation). The longer the length of the arrow, the greater the influence of the clinical factor. b) Significant associations between gut microbial taxa and clinical variables at the species level analyzed by MaAsLin (n=274). **Abbreviations**: HC, healthy controls; ITP, immune thrombocytopenia; HB, hemoglobin; PLT, platelets; WBC, white blood cells; CCA, canonical correspondence analysis; MaAsLin, multivariate association with linear model.


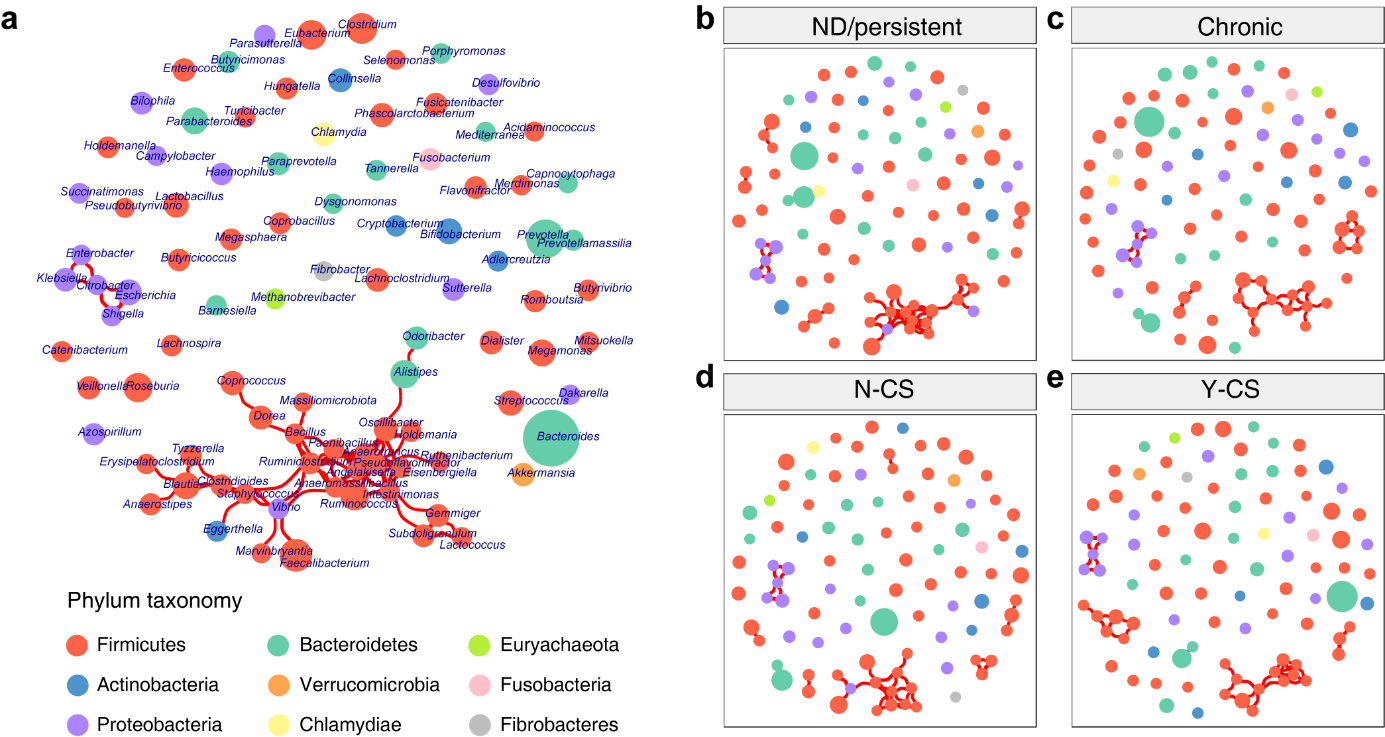


**Figure S2.** **Gut microbial community networks are constructed based on genera in ITP subpopulations.** a) The microbial co-occurrence network was deduced using Spearman rank correlations based on samples from healthy volunteers (n=62). b-c) The microbial co-occurrence network is based on samples from (b) ND/persistent (n=108) and (c) chronic (n=104) ITP patients. d-e) The microbial co-occurrence network based on samples from ITP patients who did not previously receive corticosteroid treatment (d) in the N-CS group (n=97) or did (e) in the Y-CS group (n=115). Only the correlation coefficient >0.7 with statistically significant (*p* < 0.01) connections are shown (positive correlation, red edge; Negative correlation, blue edges). Each node represents a genus, node size represents the relative abundance of the genus, and color represents the affiliated phylum. **Abbreviations**: ITP, immune thrombocytopenia; CS, corticosteroids.


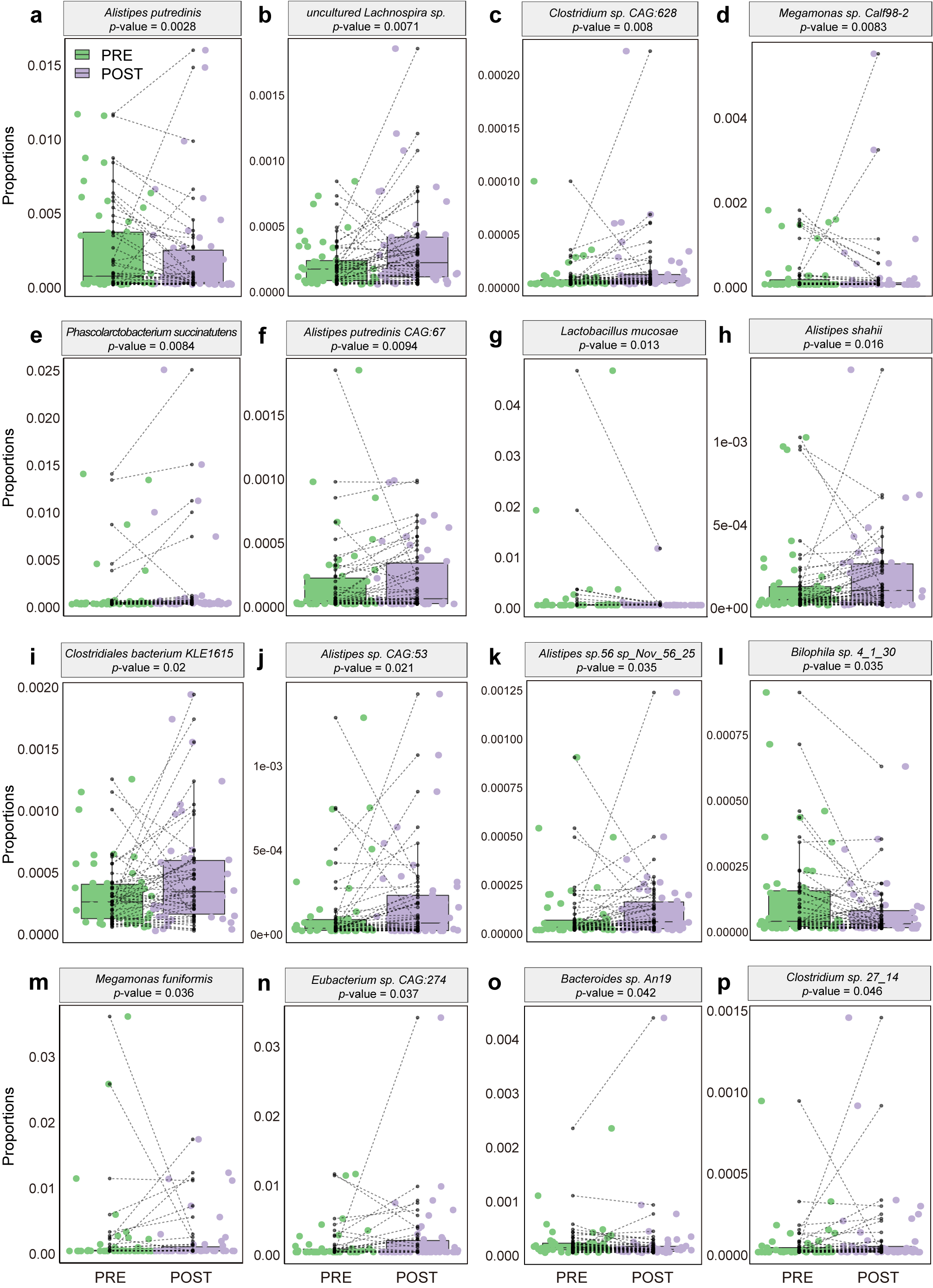


**Figure S3. Differential gut microbial species in the pre- and post-CS treatment samples of ITP.** PRE, n=46; POST, n=46. The listed 16 species were the differential species between pre- and post-CS treatment samples of ITP with *p* < 0.05 in the paired Wilcoxon test. Each box represents the interquartile range (IQR, the range between the 25th and 75th percentiles) of the relative abundance with the mid-point of the data. Dotted lines connect the points corresponding to samples collected before and after treatment from the same patient. **Abbreviations**: ITP, immune thrombocytopenia; CS, corticosteroids.


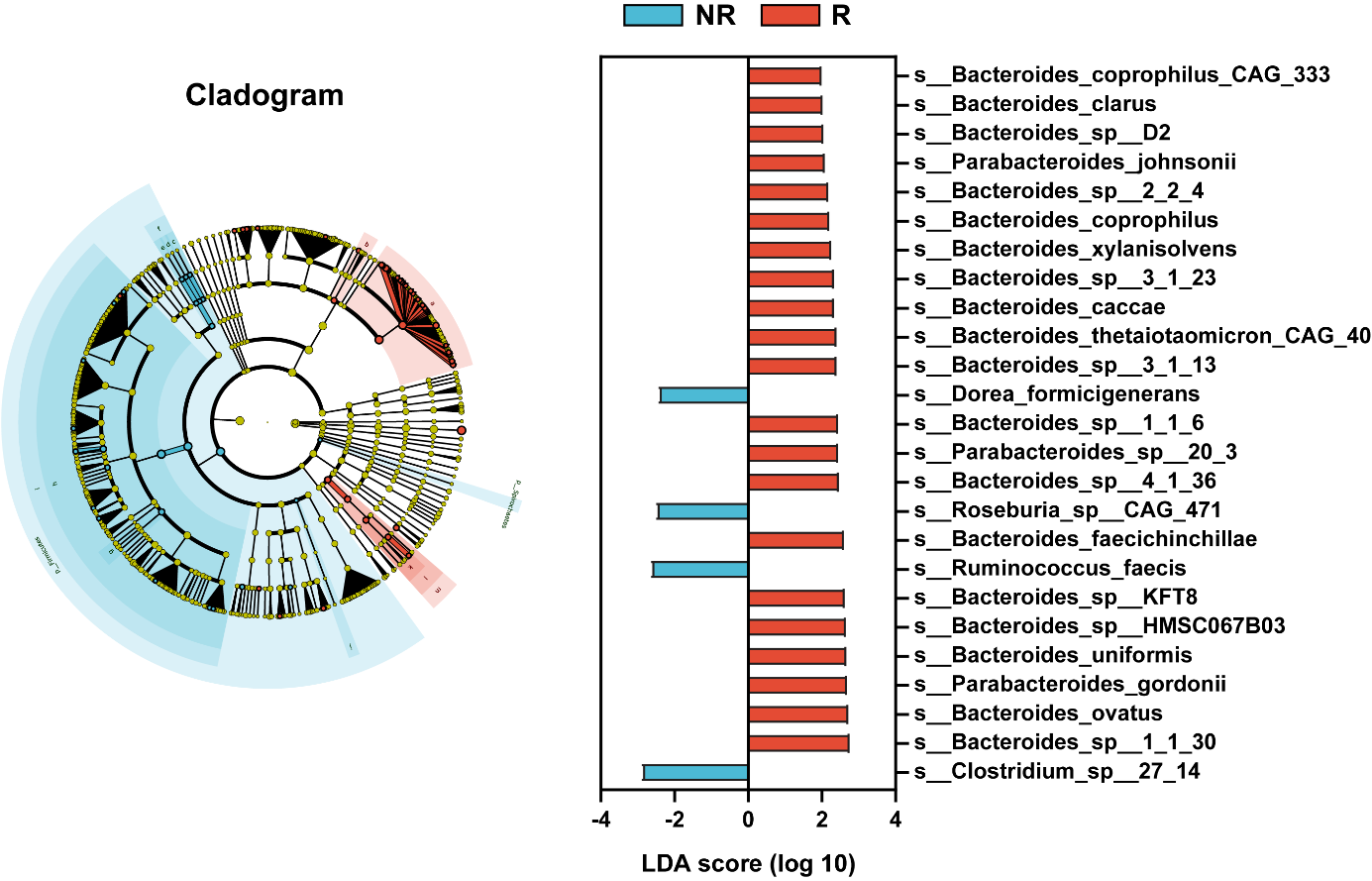


**Figure S4. LEfSe analyzed differentially abundant taxa between fecal samples from responders and non-responders.** The taxa with statistical significance (Kruskal-Wallis’s test, *p* < 0.05; LDA score > 2) were listed in the cladogram and histogram. The color represents the ITP group in which the taxa were enriched. R: responders to corticosteroid treatment (n = 71); NR: non-responders to corticosteroid treatment (n = 29). Data was shown in Supplementary Materials (Table S14). **Abbreviations**: ITP, immune thrombocytopenia; LDA, Linear discriminant analysis; LEfSe, LDA Effect Size.


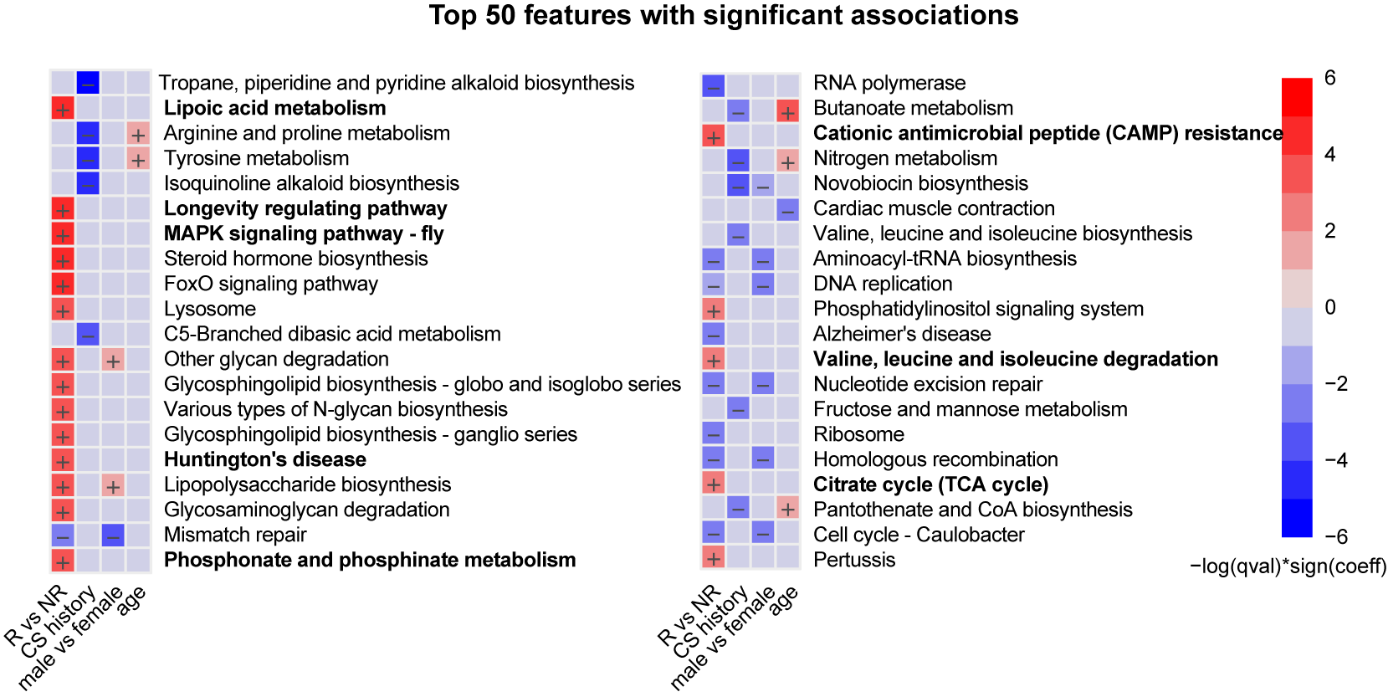


**Figure S5. Significant associations between KEGG functional profiles and clinical indices were analyzed by MaAsLin (n=100).** Data from the figures was presented in Supplementary Materials (Table S18). **Abbreviations**: CS, corticosteroid; NR, no response; R, response; MAPK, mitogen-activated protein kinases; TCA, tricarboxylic acid cycle; MaAsLin, multivariate association with linear model.

**
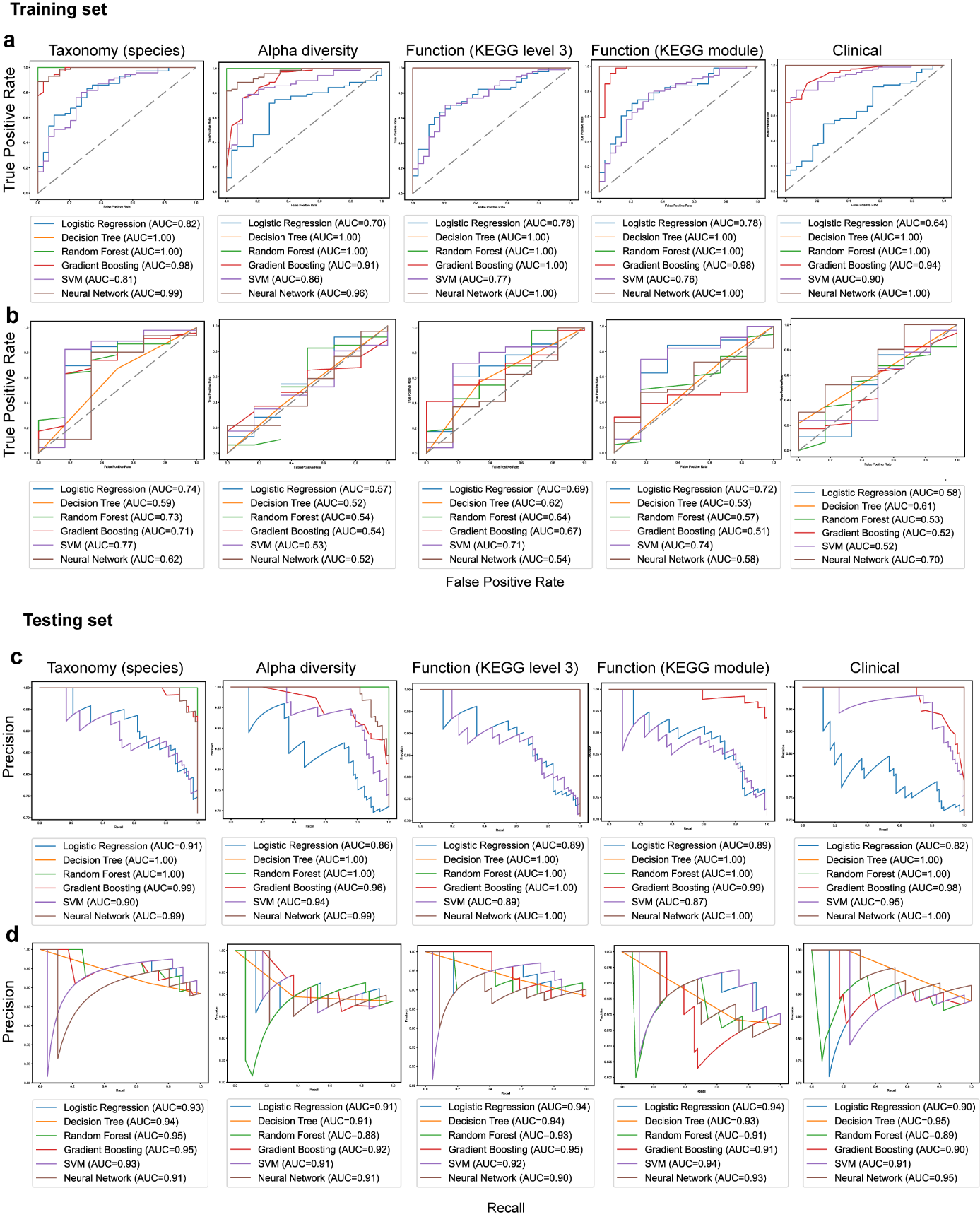
**

**Figure S6. The performance metrics of machine learning models in predicting CS responsiveness across various predictive combinations.** Training set, n=100; Testing set, n=52. Data from the figures was presented in Supplementary Materials (Table S21). **Abbreviations**: ITP, immune thrombocytopenia; CS, corticosteroids; AUC, the area under curve; SVM, support vector machines.

**
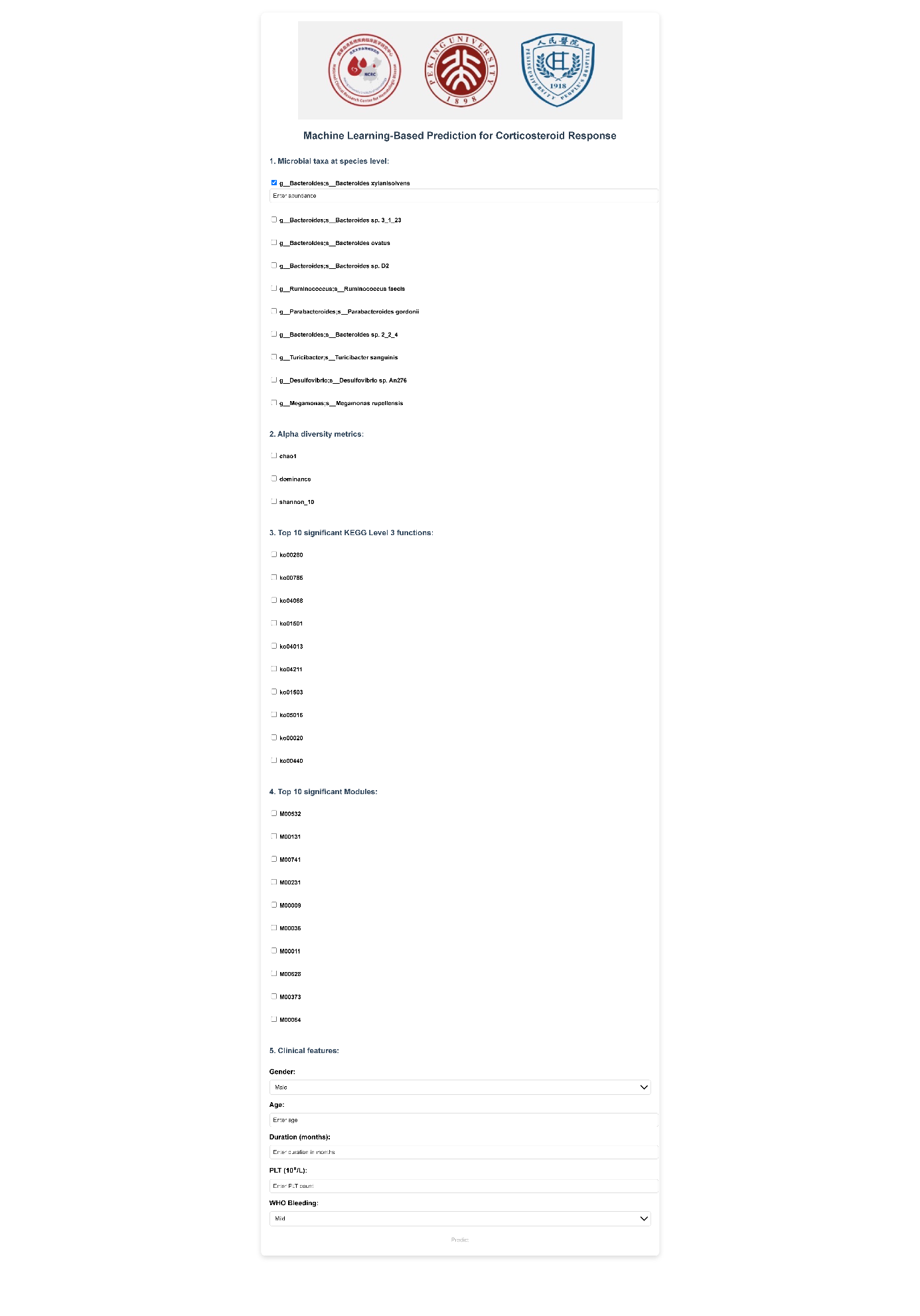
**

**Figure S7. Internet online application of prediction using clinical indicators and relative abundance results of species.**

**Supplemental Tables**

Table S1 Metadata of the 274 samples included in the study

Table S2 Summary of the 274 samples included in the study by ITP and HC groups

Table S3 Significance and importance results of clinical indices from CCA (n=274)

Table S4 Significant associations between gut microbial taxa and clinical variables at the species level analyzed by MaAsLin (n=274)

Table S5 PERMANOVA on Bray‒Curtis distance among patients with ITP regarding disease duration on order level

Table S6 Comparison of alpha diversities among patients with ITP regarding disease duration on order level

Table S7 PERMANOVA on Bray‒Curtis distance among CS subgroups of patients with ITP on order level

Table S8 Comparison of alpha diversities among CS subgroups of patients with ITP on order level

Table S9 Significant associations between gut microbial taxa and ITP subgroups at the species level analyzed by MaAsLin (n=274)

Table S10 Clinical characteristics of the longitudinal study population

Table S11 The results of the comparison between the PRE and POST groups at the species level

Table S12 Participant characteristics in development and validation cohorts

Table S13 PERMANOVA on Bray‒Curtis distance between responders vs. non-responders on order distribution

Table S14 LEfSe analysis on differentially abundant taxa between responders and non-responders

Table S15 Differential analysis on abundant taxa between responders and non-responders

Table S16 PERMANOVA on Bray‒Curtis distance between responders vs non-responders on KOs

Table S17 Differential analysis on function pathways of KEGG (level 3) between responders and non-responders

Table S18 Significant associations between gut microbial functional pathways and clinical variables analyzed by MaAsLin (n=100)

Table S19 Differential analysis on modules of KEGG between responders and non-responders

Table S20 Machine learning models for predicting CS response based on taxonomy information

Table S21 Machine learning models for predicting CS response based on different combinations

Table S22 MCC-based threshold selection in 5-fold cross-validation

Table S23 The list of clinical research centers included in Cohort 3

Table S24 Data production, quality control and gene prediction of 274 samples

**Supplementary materials**

- The STORMS checklist
- Gut Microbiota Code.py
